# Researches on the Frequency of Cancer

**Published:** 1843-10

**Authors:** 


*Researches on the Frequency of Cancer.*—Dr. Tanchou presented to the French Academy of Sciences, at its session of the 3d of July, a very elaborate memoir on the Relative Frequency of Cancer.

The frequency of diseases, said Dr. T., is in direct ratio to the susceptibility of the organs which are affected by them. When this does not occur, it is to be attributed to some accidental circumstance. Cancer does not escape this general law. But what has not yet been investigated are the order and nature of the causes of this disease. Imagining that the effects of civilization might play no small part in the production of this affection, Dr. T. consulted, with the assistance of the Prefect of the Seine, Count Rambuteau, the civil registers of that Department. 1848 quires, forming the collection from 1830 to 1840, inclusive, 11 years, were examined.

It appears that in this lapse of time there died at Paris, and the districts of Sceaux and St. Denis, 382,851 persons. Of this number, 194,735 were men; and 158,116 were women. 9118 of these died of cancerous affections; of whom 2161 were males; 6957 were women. The excess of the latter was of course 4796.*

| Deaths by Cancer. | | Deaths by Cancer. | |
|---|---|---|---|
| In 1830, | 668 | In 1836, | 837 |
| 1831, | 865 | 1837, | 778 |
| 1832, | 814 | 1838, | 803 |
| 1833, | 814 | 1839, | 887 |
| 1834, | 857 | 1840, | 889 |
| 1835, | 906 | | |
| Total, | | | 9118 |

That is, about 1.96 per cent. on the deaths of 1830, and 2.40 on those for 1840; which proves that cancer is on the increase.

---

* Under the name of cancer, Dr. T. includes not only cancerous ulcers, but schirrus, carcinoma, osteo-sarcomas, encephaloid tumours, cancers of the skin, nose, lupus, sarcoceles—in a word, all local malignant affections.

In Paris, alone, during this time.

| Deaths by Cancer. | | Deaths by Cancer. | |
|---|---|---|---|
| In 1830, | 595 | In 1836, | 728 |
| 1831, | 756 | 1837, | 674 |
| 1832, | 712 | 1838, | 703 |
| 1833, | 721 | 1839, | 779 |
| 1734, | 752 | 1840, | 779 |
| 1835, | 800 | | |
| Total, | | | 7999 |

That is to say, 2.54 per cent.; whilst in the arondissements of Sceaux and St. Denis united, there were:

| | | | |
|---|---|---|---|
| In 1830, deaths | 73 | In 1836, deaths | 109 |
| 1831, | 100 | 1837, | 104 |
| 1832, | 102 | 1838, | 100 |
| 1833, | 93 | 1839, | 108 |
| 1834, | 105 | 1740, | 110 |
| 1835, | 106 | | |
| Total, | | | 1119 |

Which gives 1.63 per cent. for the suburbs, whilst *intra muros* it was 2.54 per cent., showing that this disease is much more frequent in the capital than in its environs.

Considered with regard to age, he found the following results:

| Age. | Deaths. | Men. | Women. |
|---|---|---|---|
| From 1 to 10 years, | 23 | 9 | 14 |
| 10 to 20 | 26 | 13 | 13 |
| 20 to 30 | 231 | 62 | 169 |
| 30 to 40 | 1012 | 190 | 822 |
| 40 to 50 | 1975 | 339 | 1636 |
| 50 to 60 | 2108 | 488 | 1620 |
| 60 to 70 | 2067 | 598 | 1469 |
| 70 to 80 | 1315 | 398 | 917 |
| 80 to 90 | 335 | 62 | 273 |
| 90 to 100 | 26 | 4 | 22 |
| Total, | 9118 | 2163 | 6955 |

Examined in connection with the various organs affected, he found:

| | | | |
|---|---|---|---|
| The Uterus, | 2996 | Shoulder, | 4 |
| The Stomach, | 2303 | Throat, | 4 |
| Female Mamma, | 1147 | Ear, | 4 |
| Liver, | 578 | Pharynx, | 4 |

| | | | |
|---|---|---|---|
| Rectum, | 221 | Forearm, | 3 |
| Abdomen, (?) | 188 | Kidneys, | 3 |
| Intestine, | 146 | Parotid Gland, | 3 |
| Bladder, | 72 | Tonsils, | 3 |
| Face, | 71 | Larynx, | 3 |
| Mesentery, | 66 | Palate, | 3 |
| Ovary, | 64 | Temple, | 2 |
| Tongue, | 36 | Chin, | 2 |
| Eye, | 24 | Back, | 2 |
| Jaw, | 24 | Pancreas, | 2 |
| Brain, | 28 | Iliac Fossa, | 2 |
| Testicle, | 21 | Cœcum, | 2 |
| Lips, | 16 | Vulva, | 2 |
| Vagina, | 14 | Umbilicus, | 2 |
| Spleen, | 13 | Haunch, | 2 |
| Anus, | 13 | Cranium, | 1 |
| Esophagus, | 13 | Cerebellum, | 1 |
| Neck, | 13 | Ethmoid Bone, | 1 |
| Cheek, | 12 | Orbit, | 1 |
| Nose, | 11 | Retina, | 1 |
| Mouth, | 11 | Mastoid Process, | 1 |
| Thigh, | 10 | Back of Neck, | 1 |
| Penis, | 10 | Sternum, | 1 |
| Leg, | 9 | Pleura, | 1 |
| Thorax, | 8 | Peritoneum, | 1 |
| Axilla, | 8 | Jejunum, | 1 |
| Thyroid Gland, | 8 | Ileum, | 1 |
| Scrotum, | 7 | Female Urethra, | 1 |
| Inguinal Region, | 7 | Perineum, | 1 |
| Lung, | 7 | Scapula, | 1 |
| Colon, | 7 | Bones of the Ilium, | 1 |
| Head, | 6 | Pelvis, | 1 |
| Heart, | 6 | Sacrum, | 1 |
| Arm, | 6 | Thigh, | 1 |
| Epiploon, | 5 | No organs designated,* | 829 |
| Prostate, | 5 | | —— |
| Male Mamma, | 5 | Grand Total, | 9118 |
| Hand, | 5 | | |
| Forehead, | 4 | | |

* Under this head those cases are included which were simply marked on the register as *cancer*. Dr. T. seems to think that cancer of the breast was meant in the majority of these cases; which, added to 1147 cases of schirrus of the female mamma, and 5 in the male, would give 1981 cancers of the breasts in the two sexes.

By this statement it will be seen that there is an increase in cancerous affections, and that they are much more frequent in cities than in the country. This has already been remarked in Berlin,* and in England.†

Dr. Tanchou gives the following cases for cancer of the uterus:

In 1830, 351 cancers of the uterus; 1831, 391; 1832, 396; 1833, 398; 1834, 436; 1835, 508.

Dr. Tanchou adds that a similar comparative tabular statement between the capitals and large cities, and the rural districts, is much to be desired.

Dr. T. states that cancer is a very ancient disease of civilized life. The first example is that of Mossa, daughter of Cyrus, and wife of Cambyses, B. C. 521. What is very extraordinary is, that, according to Herodotus and others, she was cured by Demeidus, a physician of Croton, without an operation.

It is said that cancers have been found among the mummies of Egypt; and Hamon, a very distinguished veterinary surgeon, who was fourteen years in the service of Mehemet Ali, never observed cancerous affections among the native female, but occasionally among the Turkish women. Dr. Clot-Bey has made the same remark. Cancer, according to Dr. T., is like insanity, much more common in civilized countries. It has been found that in the East it is much more common among the Christians than Mahomedans. Fabricius Hildanus believed that this disease occurred more frequently in temperate climates than in warm. Rouzet says that it is very rare in Africa. The result of Dr. T.'s researches on this point leave no doubt. Dr. Bac, surgeon to the second regiment of African Chasseurs, never saw a case, even at Senegal, where he practised six years. The medical officers of the French Army are agreed on this question. Baudens, the surgeon in chief of Val-de-Grace, who enjoyed a considerable practice at Algiers for eight years, saw only in that time two or three cases. Finally, Dr. Puzin, who, in 1835, established a civil hospital ten leagues beyond the French outposts, in the midst of the Arabs, did not see a case of genuine cancer out of 10,000 patients.

Dr. Tanchou discusses finally the treatment of cancer. Having examined this disease among animals, he gives some

---

* Siebold's Journal, 1826.

† Cyclopedia of Surgery, art. Cancer.

interesting details relative to its frequency, according to age, sex, and the organ affected, He concludes thus:

1. The number of cancerous diseases seems to increase from year to year, and to be in direct ratio to the civilization of the country and of the people.

2. It is in old persons, and in women more particularly, that this malady is most to be feared. But early life is not exempt from it.

3. The organs that are most sensitive, and of glandular organization, are most obnoxious to it.

4. The cause of this disease would seem to exist in the whole economy, but more particularly in the fluids than in the solids.

5. When there is no external cause it would appear to result from a molecular organic modification, occasioned by various causes, but which, in the majority of cases, we may hope to destroy. (?)

6. In the present state of our science the treatment is only empirical, as in syphilis.

7. This treatment should include all therapeutic means, and should neither be exclusive, nor specific.

8. Finally, after twenty-two observations, which were presented to the Academy, and some scattered facts found in the science, it is not yet demonstrated that cancer is incurable in all cases. You can modify the affection, render chronic an acute case, dissipate, lessen, or render stationary the majority of primary engorgements. And it is to be hoped that we shall yet go further.—*Med. Examiner.*

